# The crystal structure of the regulatory domain of the human sodium-driven chloride/bicarbonate exchanger

**DOI:** 10.1038/s41598-017-12409-0

**Published:** 2017-09-21

**Authors:** Carolina M. Alvadia, Theis Sommer, Kaare Bjerregaard-Andersen, Helle Hasager Damkier, Michele Montrasio, Christian Aalkjaer, J. Preben Morth

**Affiliations:** 1 Norwegian Centre for Molecular Medicine, Nordic EMBL Partnership University of Oslo, Gaustadalléen 21, 0349 Oslo, Norway; 2 Institute for Experimental Medical Research, Oslo University Hospital, N-0424 Oslo, Norway; 3 Department of Biomedicine, Aarhus University, 8000 Aarhus, Denmark

## Abstract

The sodium-driven chloride/bicarbonate exchanger (NDCBE) is essential for maintaining homeostatic pH in neurons. The crystal structure at 2.8 Å resolution of the regulatory N-terminal domain of human NDCBE represents the first crystal structure of an electroneutral sodium-bicarbonate cotransporter. The crystal structure forms an equivalent dimeric interface as observed for the cytoplasmic domain of Band 3, and thus establishes that the consensus motif VTVLP is the key minimal dimerization motif. The VTVLP motif is highly conserved and likely to be the physiologically relevant interface for all other members of the *SLC4* family. A novel conserved Zn^2+^-binding motif present in the N-terminal domain of NDCBE is identified and characterized *in vitro*. Cellular studies confirm the Zn^2+^ dependent transport of two electroneutral bicarbonate transporters, NCBE and NBCn1. The Zn^2+^ site is mapped to a cluster of histidines close to the conserved ETARWLKFEE motif and likely plays a role in the regulation of this important motif. The combined structural and bioinformatics analysis provides a model that predicts with additional confidence the physiologically relevant interface between the cytoplasmic domain and the transmembrane domain.

## Introduction

The regulation and maintenance of intra- and extracellular pH homeostasis is fundamental for life. The main buffering system in humans is based on the equilibrium between HCO_3_^−^ and CO_2_ and their efficient transport across the plasma membrane of all cells^1^. The sodium-driven chloride/bicarbonate exchanger (NDCBE) is a member of the sodium-bicarbonate cotransporters (NBCs) within the solute carrier 4 (*SLC4*) gene family^2^. The NBCs include, among others, the electroneutral sodium-bicarbonate cotransporter (NCBE/NBCn2), which is mainly expressed in the brain especially in the choroid plexus^3^, and the electrogenic sodium-bicarbonate cotransporter 2 (NBCe2), which is mainly expressed in the kidney, liver and the choroid plexus^2,4^. NDCBE is electroneutral and believed to exchange one Cl^−^ for two HCO_3_^−^ and one Na^+^^2,5^. In the brain, NDCBE is primarily associated with the presynaptic vesicles of glutamatergic and GABAergic neurons^6^. Even though no human disease has been associated with NDCBE mutations, knockout mice exhibit defective regulation of Na^+^ reabsorption and reduced neuronal excitability^7,8^. In addition, low NDCBE expression levels have been reported in mouse brains in connection with chronic continuous hypoxia^9^. The primary structure of *SLC4* transporters is divided into three major domains, an N-terminal regulatory domain, a transmembrane domain containing 14 membrane-spanning helices, and a C-terminal domain often associated with binding to carbonic anhydrase^2,10,11^. The transmembrane domain is largely conserved across *SLC4* family members and shares more than 38% sequence identity with anion exchangers (AEs) and NBCs^2^. In the plasma membrane, most *SLC4* transporters are believed to form functional dimers. The molecular basis for the dimerization was recently confirmed by a crystal structure of the transmembrane dimer of AE1 (PDB ID: **4YZF**) reported by Arakawa *et al*. at 3.5 Å resolution^10^. The crystal structure did not include the cytoplasmic regulatory domains, and the organization of these with respect to the transmembrane dimer is still based on models. The primary structure of the isolated cytoplasmic regulatory domains shares lower sequence identity across the *SLC4* family, than the transmembrane regions. Based on sequences with either low or high sequence variability, the cytoplasmic domains have been divided into regions referred to as constant regions (CRs) and variable regions (VRs)^12^. The VRs mainly include the different functional and regulatory motifs of *SLC4* family members and their isoforms^13^. In addition, the VRs have been classified as intrinsically disordered regions, which are a specific dynamic type of element that lack any ordered secondary structures such as helices or strands^14,15^. The crystal structure of the cytoplasmic domain of Band 3 (cdb3) (PDB ID: **1HYN**, 2.6 Å resolution) contains a dimer interface connected by a domain-swapped β-sheet that extends into a helical segment often referred to as the ‘dimerization arm’^16^. The monomeric cytoplasmic domain, excluding the VR1, reveals a cytoplasmic N-terminal core domain consisting of the intertwined secondary structure elements CR1 and CR2. The intrinsically disordered region (VR2) forms a connecting linker between CR1 and CR2^16^. A more recent structural model of the cdb3 lacking residues 1 to 55 has been determined at 2.2 Å resolution and crystallized under near physiological conditions (pH 6.5) (PDB ID: **4KY9**). Structural comparison with the previous model at pH 4.5 shows only minor molecular differences mainly present on the domain surface^17^. Transport regulation by the *SLC4* HCO_3_^−^ transporters exerted by the N-terminal domain has been described previously^2^. However only a few publications, so far, describe that divalent metal ions like Mg^2+^ and Zn^2+^ can modulate the transport activity^18–20^. The electrogenic activity of NBCe1 was inhibited by Mg^2+^ (*K*_i_ of 0.01 mM) in whole-cell patch-clamp studies^18^, and the transport activity by AE1 was predicted to be stimulated by intracellular levels of Mg^2+^ ^19^. AE2 show partial inhibition by extracellular Zn^2+^ when expressed in *Xenopus* oocytes^20^ while equivalent concentration of extracellular Zn^2+^ had no effect on AE1 activity^20^. Here, we present the crystal structure of the truncated (ΔVR1, residues 1–39) N-terminal cytoplasmic domain from human NDCBE-D (ntcNDCBE) at 2.8 Å resolution. Structural analysis confirms the dimerization interface of previous structural models and determines that the key dimerization motif is a minimal beta-sheet that lacks the residues equivalent to the dimerization arm in cdb3. We identify and describe a novel Zn^2+^-binding site and the completely conserved HHH motif also found in the electroneutral sodium-bicarbonate transporters (NBCn1 and NCBE) surrounded by a shell of negatively charged residues on the surface of ntcNDCBE. Zn^2+^-binding properties are verified *in vitro* for ntcNDCBE and, combined with cellular assays performed on NCBE and NBCn1, this indicates a general Zn^2+^ dependence that could expand to all members of the *SLC4* superfamily.

## Results

### The crystal structure of cytoplasmic domain of NDCBE contains a dimer interface equivalent to cdb3

**Figure 1:**
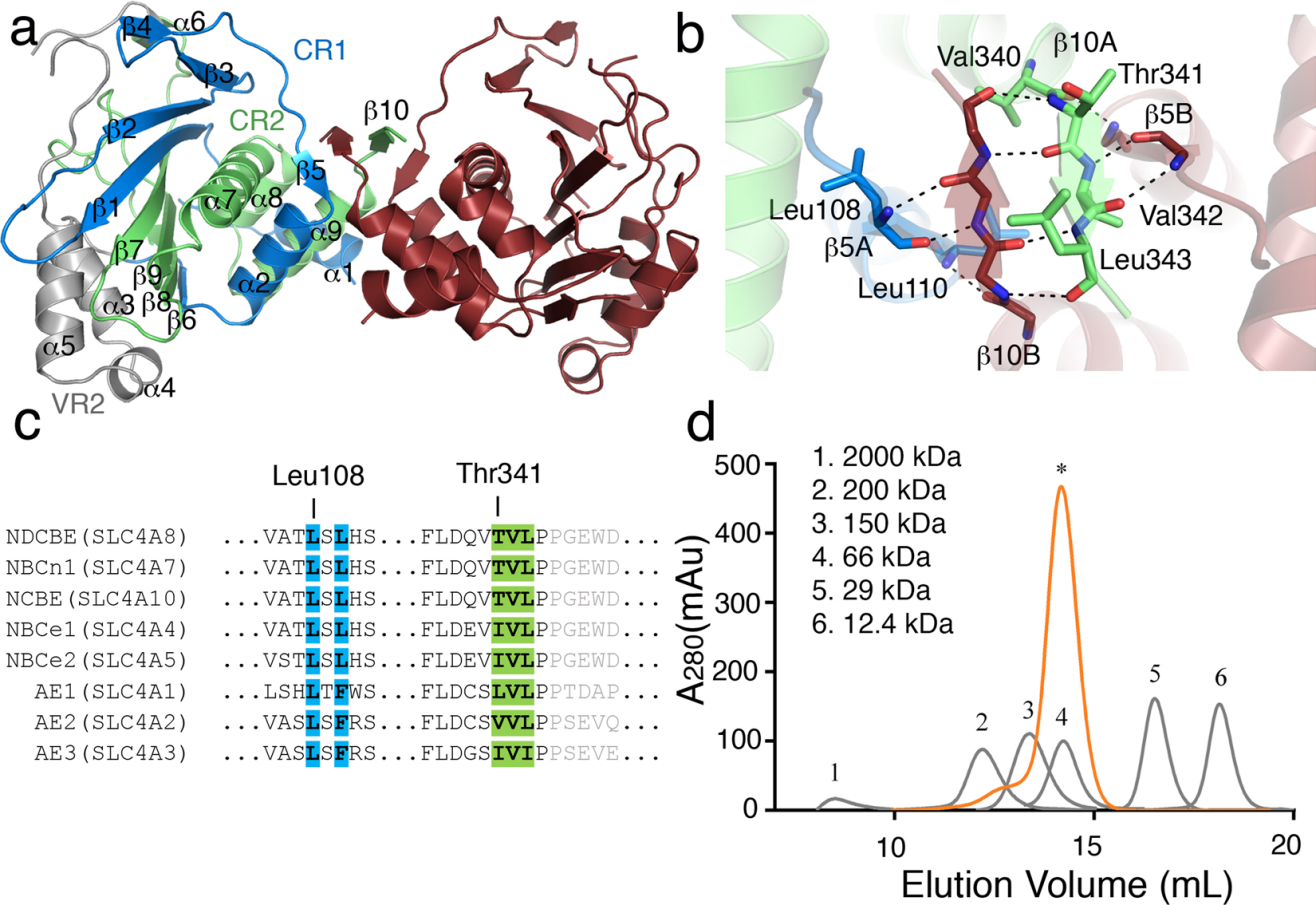
Dimerization determinants of ntcNDCBE. (**a**) ntcNDCBE is a dimer with 2-fold symmetry. The core domain of ntcNDCBE contains CR1 (light blue), VR2 (grey) and CR2 (light green). The 58 unstructured residues from Lys173 to Lys232 in VR2 are not visible in the electron density. Monomer B is shown in dark red. (**b**) Zoomed in view of the dimerization β-sheet, around 90 ° rotation along the x-axis from (**a**). The backbone of the residues of the dimerization β-sheet is shown as sticks. Only the side-chain residues from monomer A are shown. Hydrogen bonds are represented with black dotted lines. (**c**) Sequence alignment of the human N-terminal cytoplasmic domain of NBCs and AEs. Residues involved in the dimerization motif of ntcNDCBE are colour coded as in (**a**). The residues not present in ntcNDCBE are coloured grey. (**d**) Size-exclusion chromatography of 1 mg of ntcNDCBE (orange) on a Superdex 200 10/300 GL (GE Healthcare). ntcNDCBE eluted at 14.2 mL, corresponding to a dimer with an apparent MW of 80.6 kDa compared with molecular weight standards (grey).

The crystal structure of ntcNDCBE (PDB ID: 5JHO) forms a dimer in the asymmetric unit. Each monomeric ntcNDCBE forms an α-β sandwich fold by nine α-helices and ten β-strands (Fig. 1a). The main dimer interface is generated through a domain-swapped antiparallel β-sheet, including β-strands 5 and 10 (Fig. 1a and b). The dimerizing β-sheet includes four strands, two – β5 and β10 – from each monomer. Interestingly, the structural motif connects strand β5 from CR1 with strand β10 from CR2 in the domain-swapped dimerization sheet (Fig. 1a); β5 has not been previously discussed in relation to dimerization. The domain-swapped β-sheet is stabilized by backbone hydrogen-bonding interactions between residues Thr341 and Leu343 from β10 in each monomer, as well as backbone hydrogen bonds by Leu108 (β5) with Val342 (β10) and Leu110 (β5) with Val340 (loop between α9 and β10) on the adjacent sides (Fig. 1b). The residues of the β-sheet are related by a two-fold symmetry between the two subunits and are highly conserved throughout the family (Fig. 1c) as L[S/T][L/F] and [T/V/I/L]V[L/I]P motifs. In addition to what was proposed for cdb3 by Zhang *et al*.^16^, the dimerization of ntcNDCBE is only dependent on the backbone interactions of the four-membered β-sheet and the possible additional hydrophobic interactions between Leu108, Leu110, Phe114, Val337, Leu340 and Val342 from each monomer. The initial dimerization interface proposed for cdb3 included an α-helix – located downstream of β10, residues 327 to 348 in AE1 (PDB ID: **1HYN**) – as the dimerization arm, which was claimed to be important for dimerization^16^. The buried area between the 1HYN subunits is of 2500 Å, while ntcNDCBE has a buried area of 870 Å (calculated with the PDBePISA server). However, the molecular interactions of these α-helices are not symmetric, as one would expect for a dimer interface^21^; we therefore propose, based on the present study, that the dimerization arm only plays a minor role in stabilizing the dimer interface (supplementary Figure S1).

### The ntcNDCBE forms a dimer in solution

Most NBCs are known to form dimers or tetramers in the plasma membrane under physiological conditions^22^. This arrangement would naturally lead to a high local concentration of single cytoplasmic domains. High local concentrations also occur during crystallization. However, to establish whether ntcNDCBE forms a dimer in solution at lower concentrations, the construct was subjected to size-exclusion chromatography (SEC), and eluted at volumes corresponding to the hydrodynamic volume of a dimer (Fig. 1d).

### Structural comparison of cdb3 with ntcNDCBE

**Figure 2:**
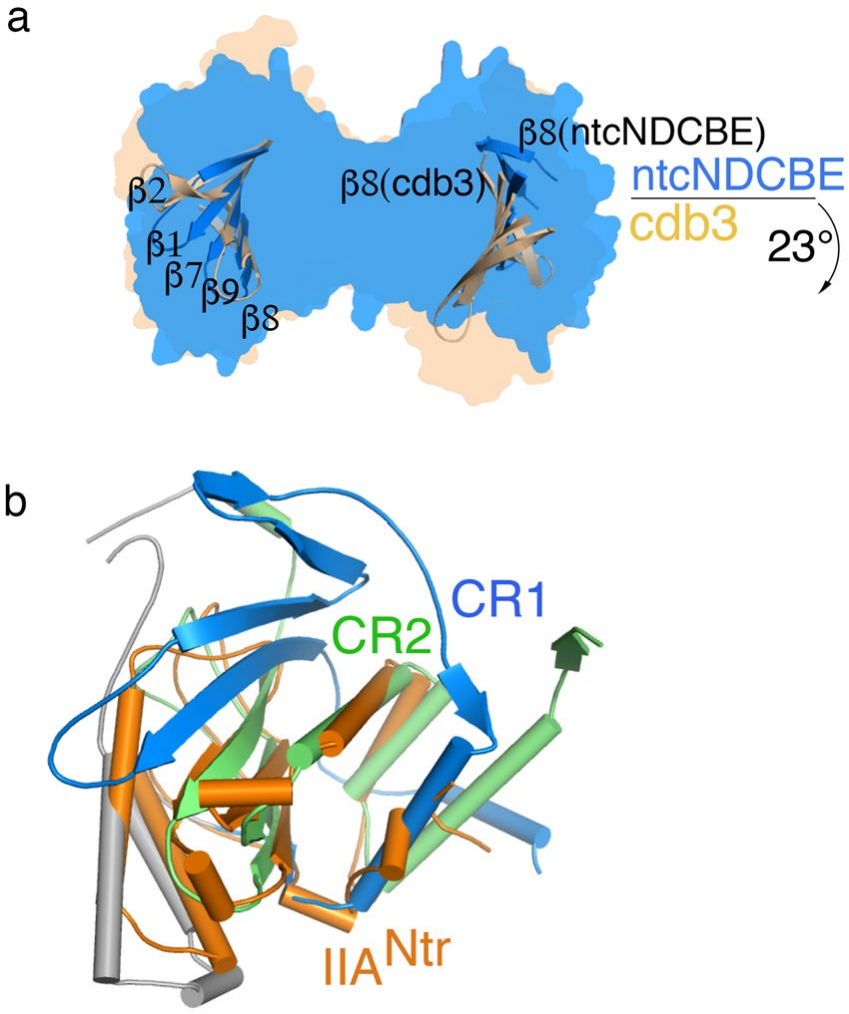
Structural comparisons of ntcNDCBE. (**a**) Superimposition of the central β-sheet of ntcNDCBE (chain A, blue) with the corresponding amino acids from cdb3 (chain P, light brown) with an RMSD of 0.82 Å. The relative movement of chain B of ntcNDCBE compared with chain Q of cdb3, showing a pivotal rotation of 23°. Only the central β-sheet of both structures is shown. The surface representation of each structure is shown in the background. (**b**) Superimposition of CR2 from ntcNDCBE chain A with E. coli IIA^Ntr^ (PDB ID: 1A6J, Chain A), showing an RMSD of 2.25 Å. IIA^Ntr^ is coloured orange.

The monomeric structures of ntcNDCBE and cdb3 (PDB ID: **1HYN**) share an overall similar fold (RMSD of 0.85 Å, calculated from C_α_ atoms). However, dimers of these two structures superimpose poorly despite the equivalent dimer interface, although they clearly show a pivotal movement between each monomer. By superimposing the central β-sheet (β1-3, β6-9) of the individual monomers of cdb3 (chain P) and ntcNDCBE (chain A), a pivotal movement of ~23° is observed between each monomer, while the central dimerization domain-swapped β-sheet (β5, β10) remains intact. This pivotal freedom hints that dynamic flexibility is allowed between the two monomers (Fig. 2a) but likely restrained to a “butterflywing-like flapping” motion with the dimerization motif centred between the monomers. Both cdb3 and ntcNDCBE were crystallized under similar pH conditions. Previous reports observed no significant pH dependent rearrangement of monomers for cdb3 obtained at pH 6.5 (PDB ID: **4KY9**).

### Structural homology of bacterial IIA^Ntr^ with ntcNDCBE

Nitrogen regulatory IIA proteins (IIA^Ntr^) are a specific class of bacterial enzymes that belong to the nitrogen-metabolic phosphotransferase system and have high structural homology with cdb3^2^, particularly *E. coli* IIA^Ntr^ (PDB ID: **1A6J**, ref.^23^). These proteins function as cytosolic regulators of membrane K^+^ and sugar transporters^24^. The fold of the cytoplasmic domain in the *SLC4* family is unique; however, it does show some structural homology to IIA^Ntr^, as described previously^2^. The lowest RMSD score from the structural alignment was achieved by aligning all the residues of IIA^Ntr^ with CR2 (α7-9 and β7-9) from ntcNDCBE (Fig. 2b). However, this structural homology arises predominantly from CR2 and parts of VR2 (α3,5) in ntcNDCBE, indicating that the sequence conservation observed in CR2 most likely reflects a set of residues that are needed to maintain the structural scaffold of the domain and that the functionality of ntcNDCBE (and the other NBCs) is therefore specific to CR1, VR2 and VR1.

### Metal-binding properties of NBCs

**Figure 3:**
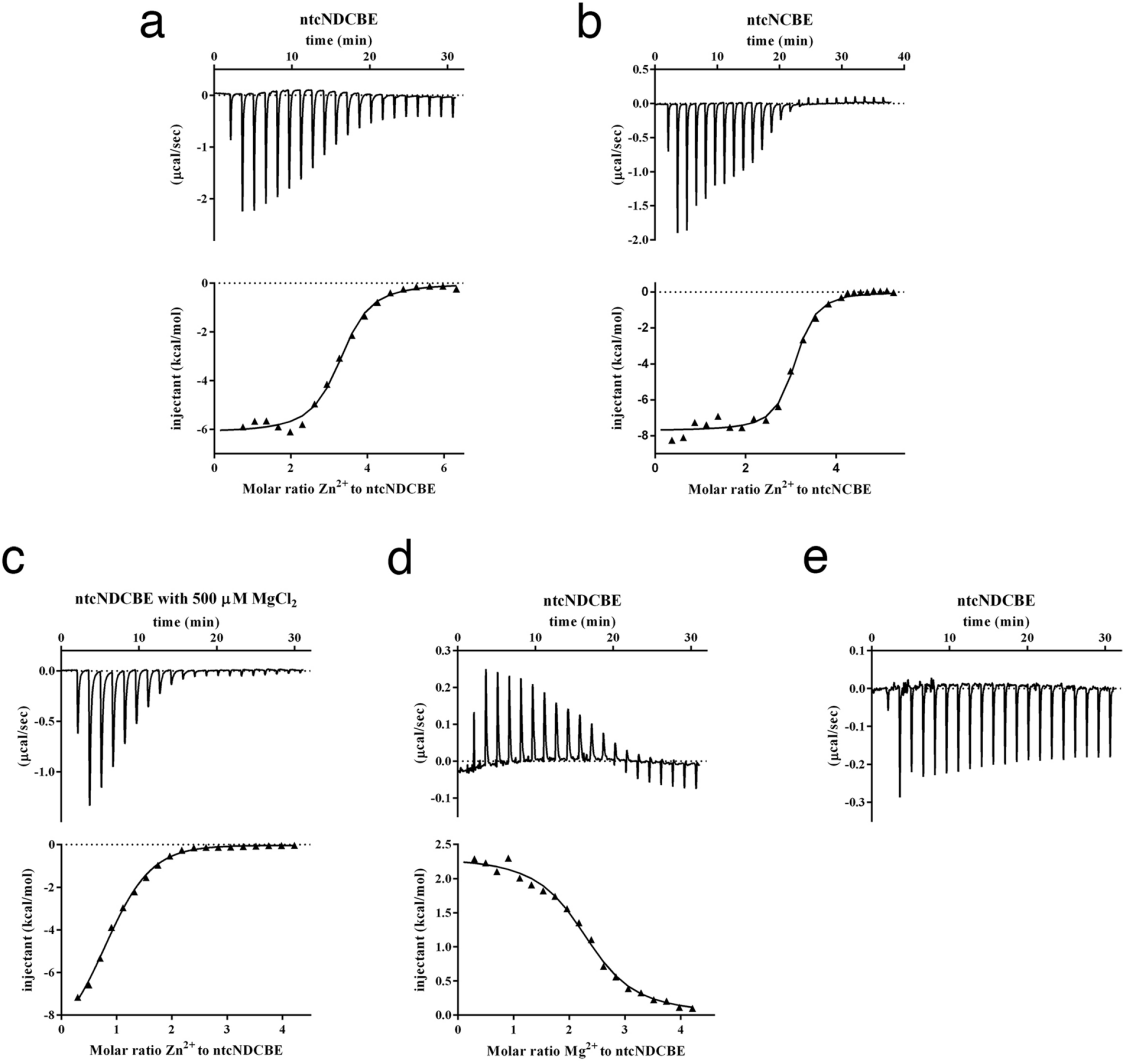
The cytoplasmic domain of NBCs has divalent metal-binding properties. (**a**–**e)** Top: the heat released/absorbed upon metal injection. Bottom: the integrated heat data and corresponding binding isotherm (black line). ITC measurement of ZnCl_2_ titration into **(a)** ntcNDCBE. Binding model: one set of sites (n = 3.2, *K*_D_ = 2.3 µM). **(b)** ntcNCBE. Binding model: one set of sites (n = 3.0, *K*_D_ = 0.7 µM). **(c)** ntcNDCBE in the presence of 500 µM MgCl_2_. Binding model: sequential binding sites (n = 2, *K*_D_ = 1.1 µM and 14.5 µM). **(d)** ITC measurement of MgCl_2_ titration into ntcNDCBE. Binding model: one set of sites (n = 2.3, *K*_D_ = 4.4 µM). **(e)** ITC measurement of CaCl_2_ titration into ntcNDCBE. No binding isotherm could be fitted.

Based on the observation that ntcNDCBE has a distinct set of histidine residues clustered on the surface, the metal-binding properties of ntcNDCBE were tested by titrating the domain with Zn^2+^ via isothermal titration calorimetry (ITC). Each injection produced an exothermic reaction that decreased in magnitude with subsequent injections. The affinity of ntcNDCBE for Zn^2+^ was determined to originate from three equivalent binding sites, with an approximate *K*_D_ of 2.3 µM and experimental thermodynamic parameters ΔH = −6130 ± 110.4 cal/mol and ΔS = 5.24 cal/mol/deg (Fig. 3a). To evaluate whether Zn^2+^ binding is a general feature among NBCs, equivalent Zn^2+^ titrations were performed with ntcNCBE (Fig. 3b). Three equivalent Zn^2+^-binding sites are present in ntcNCBE with affinities estimated to be in the low micromolar range and thermodynamic parameters of ΔH = −7711 ± 112.0 cal/mol and ΔS = 2.40 cal/mol/deg (Fig. 3b).

#### Two Zn^2+^-binding sites in ntcNDCBE

To evaluate the specificity of the Zn^2+^-binding sites in ntcNDCBE, an equivalent experiment was repeated in the presence of 500 µM MgCl_2_. The resulting titration curve (Fig. 3c) suggests that only two metal-binding sites are specific to Zn^2+^ and that the cytoplasmic domain also is capable of binding Mg^2+^. The specific Zn^2+^-binding sites present in ntcNDCBE appear to be different and allosteric, with affinities in the low micromolar range. The thermodynamic parameters determined for the first binding site were ΔH = −7709 ± 268 cal/mol and ΔS = 1.36 cal/mol/deg, and those for the second binding site were ΔH = −3815 ± 284 cal/mol and ΔS = 9.34 cal/mol/deg (Fig. 3c).

#### ntcNDCBE has two Mg^2+^-binding sites

ITC measurements with Mg^2+^ revealed two equivalent high-affinity Mg^2+^-binding sites in ntcNDCBE (Fig. 3e). The reaction between ntcNDCBE and Mg^2+^ is endothermic, with thermodynamic parameters of ΔH = 2330 ± 44.50 cal/mol and ΔS = 32.3 cal/mol/deg (Fig. 3d). In a control experiment, titration with Ca^2+^ showed no measurable interaction with ntcNDCBE (Fig. 3e).

### Conserved Zn^2+^-binding motif in NBCs

**Figure 4:**
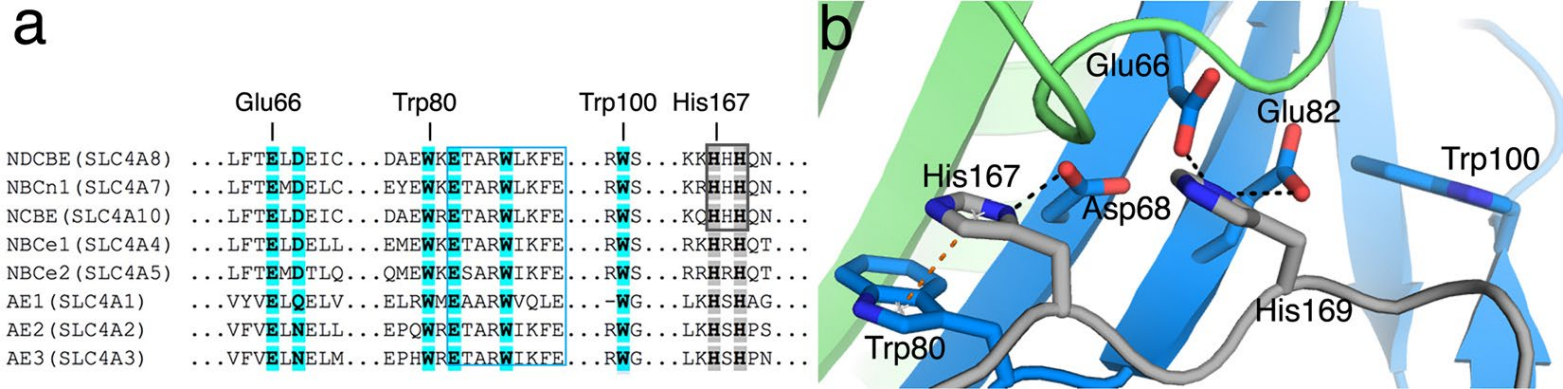
Putative Zn^2+^-binding site in ntcNDCBE. (**a**) Fraqmented sequence alignment of the N-terminal cytoplasmic domain of NBCs and AEs. The conserved ETARWLKFEE motif, proposed to be part of a substrate channel, is marked by a blue box. The residues are coloured as in Fig. 1a. (**b**) The ntcNDCBE structure: The H167-X-H169 Zn^2+^-binding motif, the secondary shell of negative residues and the three Trp residues of the construct are shown as sticks. Hydrogen bonds are represented as black dotted lines and π-stacking interactions in orange.

**Figure 5:**
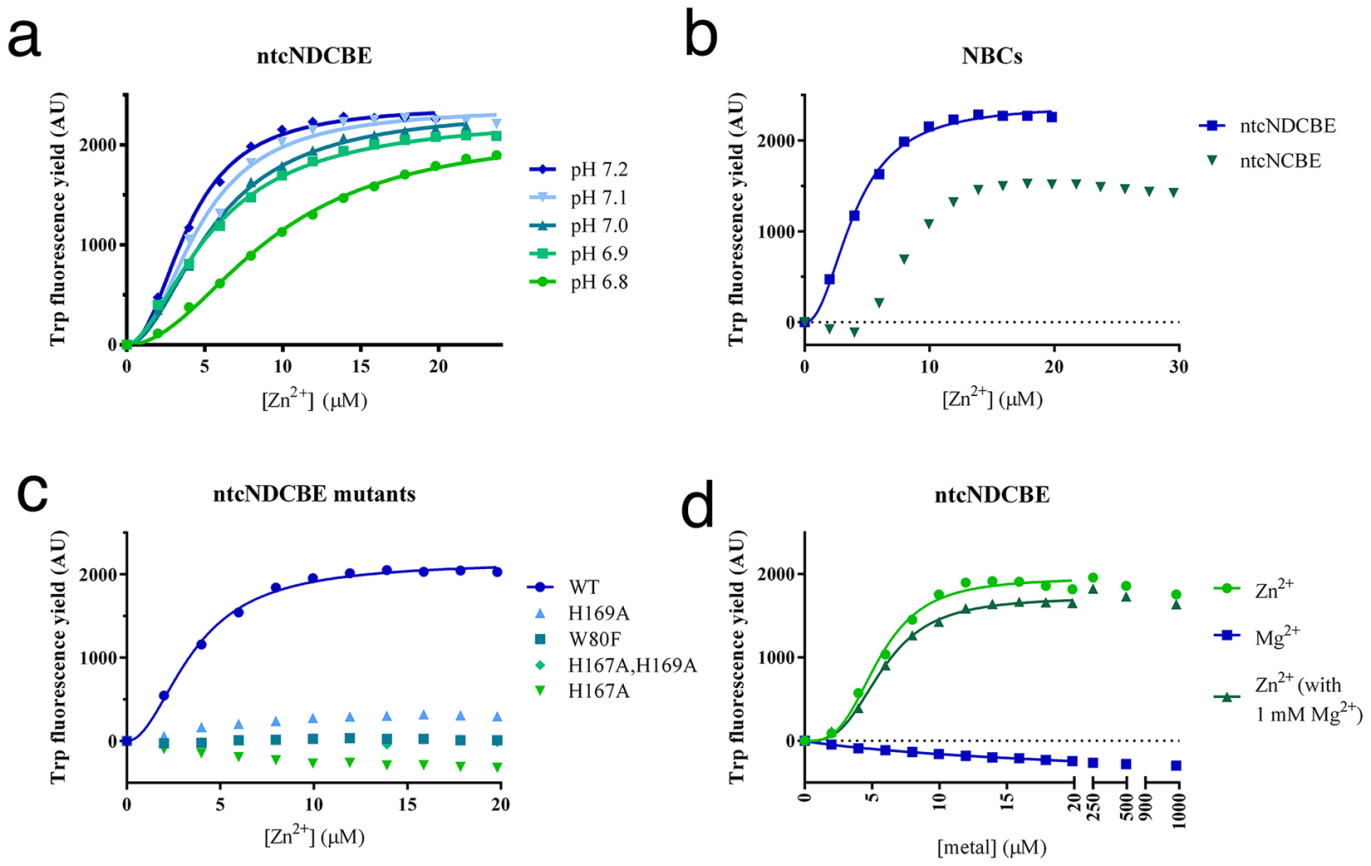
Effect of Zn^2+^ on the tryptophan fluorescence yield of NBCs. (**a**) Zn^2+^ titration into ntcNDCBE at different pH values. Individual runs are shown in Supplementary Figure S2. (**b**) Zn^2+^ titration into, ntcNCBE at pH 7.2. The Trp fluorescence yield increases faster in ntcNDCBE when exposed to Zn^2+^ as compared with ntcNCBE. Individual runs are shown in Supplementary Figure S4. (**c**) Zn^2+^ titration into the different ntcNDCBE constructs at pH 7.2. The ntcNDCBE mutants show little response to Zn^2+^ titration. Individual runs are shown in Supplementary Figure S2. (**d**) Mg^2+^ and Zn^2+^ (in the absence and presence of 1 mM Mg^2+^) titration into ntcNDCBE at pH 7.2. Individual runs are shown in Supplementary Figure S2. The fits (lines) were calculated with equation (2).

Structural identification of the divalent metal binding sites was attempted extensively by soaking and co-crystallization experiments. The experiments were severely hampered by the crystal conditions, as we were only able to form crystals at pH 4.4. At this pH, surface exposed histidines (pKa ~ 6.5) are protonated and thus unable to form a complex with any metal ions. Attempts to increase the pH after the crystals had formed or fast soak high concentrations of Zn^2+^ into the crystals reduced the diffraction quality of the crystal dramatically and made structural interpretation impossible. The highly conserved HXH (H167-X-H169) motif, which has also been found in other Zn^2+^-binding proteins^25^, was found surface-exposed on the structure of the cytoplasmic domain of NDCBE (Fig. 4a). In the human electroneutral sodium-bicarbonate cotransporters, represented by NCBE, NBCn1 and NDCBE, this motif is conserved as three histidine residues (HHH) (Fig. 4a). The HXH motif in ntcNDCBE is surrounded by a secondary shell of negatively charged residues (Fig. 4b). Sequence analysis revealed that residues from this putative site, including Glu66, Glu82, His167 and His169, are conserved throughout the *SLC4* family (Fig. 4a), whereas residue Asp68 is only conserved in the NBCs. In the AEs, either the polar residues glutamine or asparagine is present at the equivalent position to Asp68, which suggests a conserved structural feature. The ntcNDCBE structure shows that one, of the three tryptophan residues, is in close molecular proximity to the identified H167-X-H169 motif (Fig. 4b) and thus indicates that tryptophan fluorescence might be applicable to probe the structural changes that occur upon Zn^2+^ binding at this site. Indeed, titrating ZnCl_2_ into ntcNDCBE induced a strong (~100%) increase in tryptophan fluorescence as a function of Zn^2+^ concentration (Fig. 5a). To confirm the validity of this approach, we titrated Ca^2+^ into ntcNDCBE as a negative control (Supplementary Figure S2f). The resulting fluorescence binding data is in agreement with the ITC results showing no interaction. The effect observed by titrating Zn^2+^ into ntcNDCBE indicates that an interaction modifies the nearby environment of one or more tryptophan residues, the most likely candidate being Trp80. Since the Zn^2+^-binding site would depend on the protonation state of the histidine residues, the titrations were repeated at different pH values, and as expected shows lower binding affinity at lower pH values as pH approaches the theoretical pKa values of histidine (Fig. 5a). The sigmoidal shape of the curves indicates a cooperative binding mode. As a consequence, all curves were fit by the Hill equation and predicts a Hill coefficient of ~2. The binding curves obtained through this indirect approach also point to an affinity in the low micromolar range (Supplementary Table S2). To exclude the possibility that the increase in fluorescence was caused by the protein unfolding or aggregating, we titrated EDTA into ntcNDCBE in the presence of saturating Zn^2+^ concentrations (Supplementary Figure S3). The chelation of Zn^2+^ from ntcNDCBE by EDTA also shows cooperativity and restores the initial tryptophan fluorescence, indicating that the reaction is reversible. The Hill coefficients derived from the repressive Hill equation are above 5; however, the actual value of the Hill coefficient cannot be determined, as the system is already oversaturated with Zn^2+^. Zn^2+^ titrations were also performed with ntcNCBE. Although this construct also showed an increase in tryptophan fluorescence (Fig. 5b), the increase in fluorescence yield is moderate and only detectable after a 5-fold molar excess of Zn^2+^ has been added. This result indicates that the observed fluorescence yield is caused by the combined effect of multiple Zn^2+^ ions binding sporadically to the surface of the construct. As a result, these experiments were not pursued further. To confirm that the observed Zn^2+^-binding was specifically due to the H167-X-H169 motif, the tryptophan fluorescence experiments were repeated using ntcNDCBE single mutants (H167A, H169A and W80F) and a double mutant (H167A, H169A). A strong reduction in tryptophan fluorescence yield was observed for all mutants (Fig. 5c), suggesting that His167 and His169 may indeed be the direct binding residues and that both may be essential for Zn^2+^ binding. The purity and monodispersity of the mutants in solution were equivalent to those of WT, as assessed by SDS-PAGE and SEC (Supplementary Figure S5). The strongly reduced tryptophan fluorescence yield of the W80F mutant (Fig. 5c) indicate that this tryptophan residue is the one being dequenched upon Zn^2+^ addition. In the WT protein, Trp80 is engaged in a π-π stacking with His167 (Fig. 4b), which is likely rearranged upon Zn^2+^ binding. To confirm that the H167-X-H169 motif presents a unique Zn^2+^ site, Mg^2+^ was titrated against ntcNDCBE. The resulting titration curves (Fig. 5d) show that, unlike Zn^2+^, Mg^2+^ does not affect the tryptophan fluorescence profile of ntcNDCBE, both in the absence and presence of increasing micromolar concentrations of Zn^2+^, confirming that this site is specific to Zn^2+^.

### Zn^2+^ reduces NBC activity in mammalian cells

**Figure 6:**
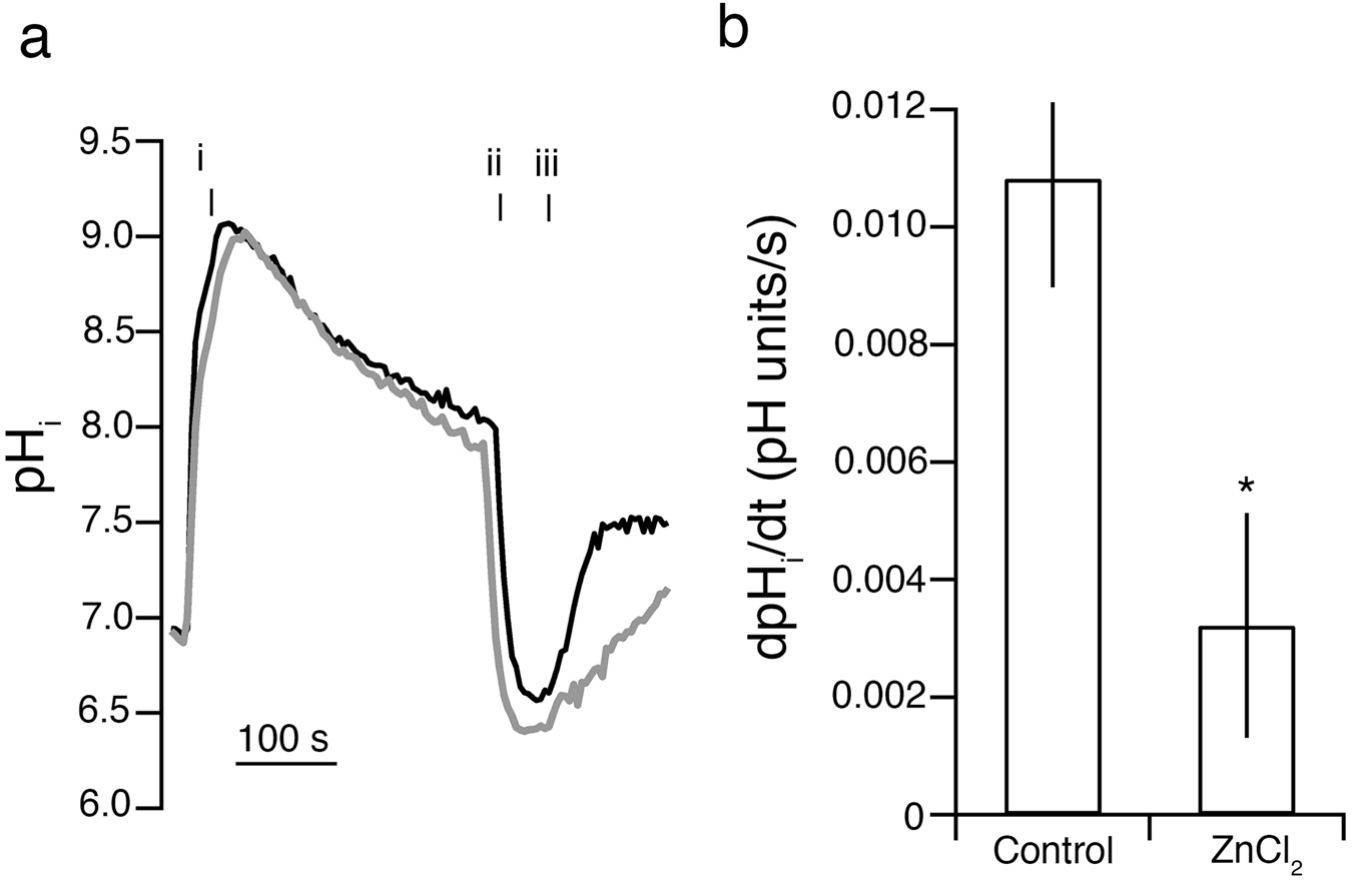
pH measurements performed in *slc4a10*-transfected cells. (**a)** Na^+^ and HCO_3_^−^ dependent pH_i_ recovery following intracellular acidification is reduced by 10 uM ZnCl_2_. Left: Intracellular pH recordings in *slc4a10* transfected cells. After a baseline pH_i_ measurement in HBS cells were acidified using an NH_4_Cl prepulse (i) followed by a washout in a Na^+^ free BBS (ii). After a short stabilization, the pH_i_ recovery was determined after the addition of Na^+^ containing BBS (iii). The recordings were performed in the absence (black line) and presence (grey line) of 10 µM ZnCl_2_ added to the BBS. **(b)** Mean values ± SEM for net acid extrusion rate in the *slc4a10* transfected cells.

To investigate the physiological effect of Zn^2+^ on NBC activity on intracellular pH regulation in mammalian cells two setups were investigated. First, the effect of Zn^2+^ on isolated *slc4a10* activity was determined in NIH-3T3 fibroblasts stably transfected with *slc4a10*. The Na^+^ and HCO_3_^−^ dependent NBC activity (dpH_i_/dt) in the cell line was reduced by 70% in the presence of 10 µM ZnCl_2_ (n = 5 (control) and n = 4 (ZnCl_2_), p = 0.0253, unpaired t-test, Fig. 6a). The experiments were performed in the presence of 10 µM EIPA which blocks NHE activity and thus represents the effect on isolated *slc4a10* activity. Furthermore, the pH recovery in isolated rat mesenteric small arteries (which reflects the combined effect of electroneutral Na^+^ dependent HCO_3_^−^ influx and Na,H-exchange activity^26^) was 0.019 ± 0.002 sec^−1^ (n = 5) and 0.013 ± 0.001 sec^−1^ (n = 5), in the absence and presence of Zn^2+^, respectively (n = 5, p < 0.05 paired t-test).

### Modelling of full-length NBC based on surface conservation of ntcNDCBE

**Figure 7:**
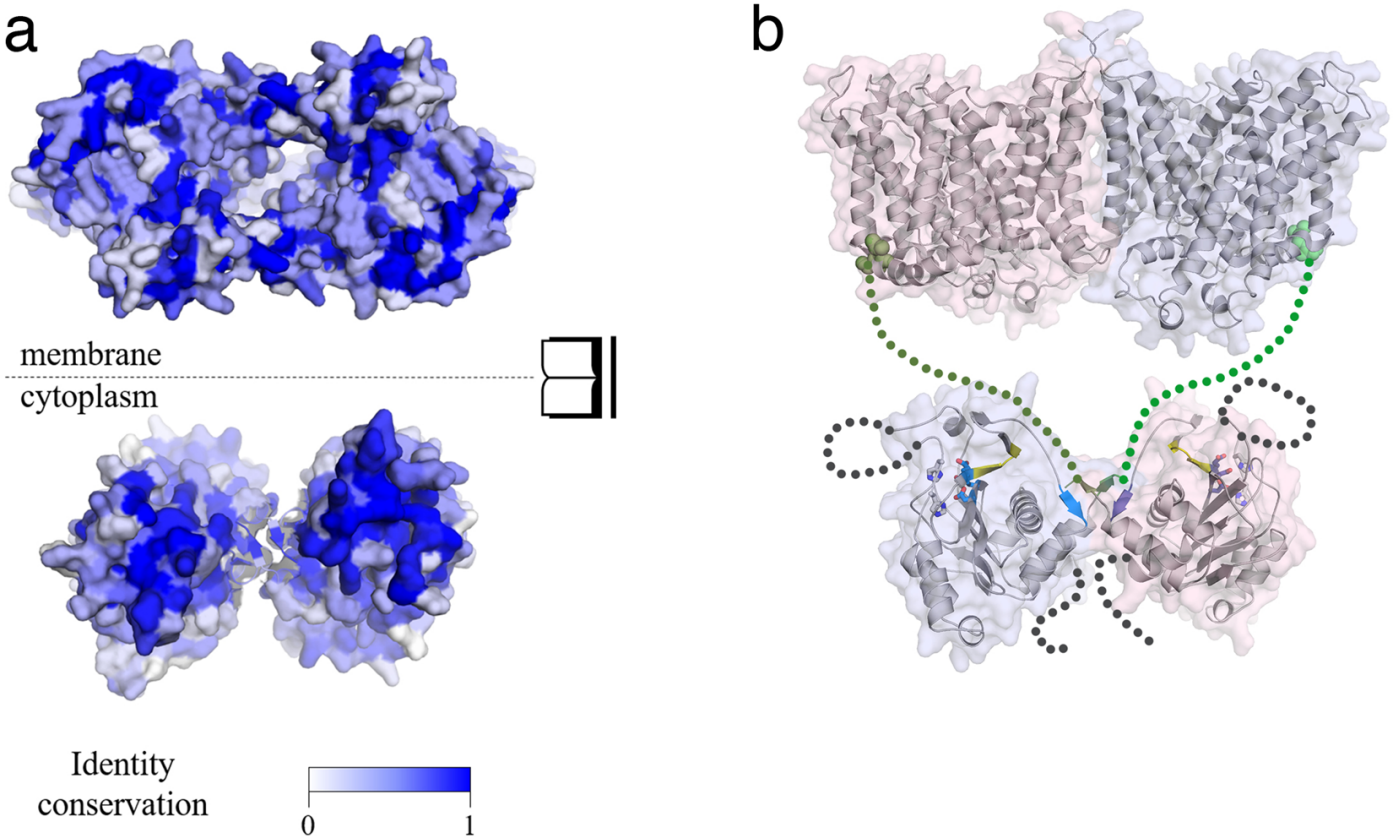
Orientation of ntcNDCBE towards the membrane. (**a)** Top: Sequence conservation of the transmembrane domain of NBCs and AEs mapped onto the transmembrane domain structure of AE1 (PDB ID: 4YZF), as viewed from the cytoplasm. Dark blue indicates high conservation. Bottom: Sequence conservation of the NBC and AE N-terminal cytoplasmic domains mapped onto the structure of ntcNDCBE, as viewed from the membrane. **(b)** Model of the structure of NDCBE. The ntcNDCBE is shown as a grey cartoon in either a blue (monomer A) or a pink (monomer B) surface. The ETARWLKFEE motif is coloured yellow. The dimerization β-sheet and the identified Zn^2+^-binding site are coloured as in Fig. 1a. Resudies in the identified Zn^2+^-binding site is shown as sticks. The transmembrane domain of AE1 is shown as a grey cartoon in either a pink (monomer A) or a blue (monomer B) surface. The first two residues of the transmembrane domain are shown as green coloured spheres, according to which N-terminal cytoplasmic monomer is bound. The intrinsically disordered VRs are represented as dotted lines. The cytoplasmic domain is not represented.

Currently, no structural information on any full-length *SLC4* member is available. The recent crystal structure of the AE1 transmembrane domain only allows speculation with regard to which surface of the cytoplasmic domain dimer face the transmembrane dimer. It is common for most proteins that the core is higher conserved compared to the surface-exposed regions^27^. The conserved surface residues that form structural patches hint at functional importance. Earlier in this paper, we presented evidence that the dimer interface found in the cdb3 dimer is also present in the here-described ntcNDCBE dimer. The presented structural evidence thus indicates that the dimer interface is indeed physiologically relevant. The dimers all form a two-fold rotational axis that is perpendicular to the β10 A/β10B sheet, leaving only two surfaces in the dimer with conserved residues facing the same direction, as described for typical homodimeric proteins such as superoxide dismutase and interleukin 10^28^. In ntcNDCBE, one of these surfaces clearly displays patches of higher conservation compared with the other (Supplementary Figure S6). We therefore propose that the most conserved surface forms a direct interaction with the transmembrane domain (Fig. 7a) to logically unite the conserved core to complete both the cytoplasmic and transmembrane domains. This assumption allows the domain orientation and architecture of the complete NDCBE transporter to be confidently modelled (Fig. 7b).

## Discussion

Here, we present the first structural data on the cytoplasmic domain of NDCBE at 2.8 Å resolution. This is the highest resolution structure yet reported for the regulatory cytoplasmic domain of an electroneutral NBC. The overall fold of ntcNDCBE is equivalent to the previously described cytoplasmic domain of the anion exchanger, Band 3. However, the cytoplasmic domain was crystallized without the previously proposed amino acid region equivalent to the dimerization arm. The dimerization motif in ntcNDCBE contains a VTVLP motif and form a small domain that is swapped by β-strands 5 and 10 to form a four-stranded β-sheet. The dimer interfaces of ntcNDCBE and cdb3 reveal that the VTVLP motif is in a hinge region and indicate that a significant pivotal movement is allowed between cytoplasmic domain monomers. The dimer is further stabilized by a patch of hydrophobic residues that form symmetric interactions between chains A and B, as is often observed at dimer interfaces^28^. The ntcNDCBE was shown to preferentially form dimers in solution. The observation of an equivalent dimer interface strongly supports the physiological relevance of the four-stranded dimerizing β-sheet. These residues are conserved in all AEs and NBCs, including both the electroneutral and electrogenic members of the SLC4 family.

Variations in intracellular Zn^2+^ levels are currently being investigated for their role in regulation and signalling events, notably at synapses^29,30^. Regulatory Zn^2+^-binding sites are well characterized and often consist of histidines and negatively charged residues. The latter are also usually present in a secondary shell surrounding the binding pocket^25,31^. The regulation of *SLC4* transporters by extracellular Zn^2+^ has been observed for AE2. The identified Zn^2+^-binding site is thought to be located in an extracellular loop; however, applying 1 mM extracellular Zn^2+^ might also interfere with intracellular Zn^2+^ concentrations^20^. To the best of our knowledge, no studies have reported the modulation of *SLC4* members by intracellular Zn^2+^ through interactions with the cytoplasmic domain. The cytoplasmic domain of *SLC4* transporters is only present in animals, adding to the common perception of an advanced regulatory role of the overall N-terminal cytoplasmic domain in higher organisms^12^. Our data reveal multiple Zn^2+^-binding sites in ntcNDCBE and ntcNCBE, all with affinities in the low micromolar range. By combining mutational studies with tryptophan fluorescence and ITC, we identified a novel, specific Zn^2+^-binding site in the structure of ntcNDCBE. The H167-X-H169 motif is highly conserved in all NBCs and AEs. This Zn^2+^-binding site is therefore likely to be a general feature of NBCs and AEs. Furthermore, this site is positioned near the proposed cytoplasmic and transmembrane domain interface, proposed by the model in this study and in close proximity to the putative substrate channel proposed by Chang *et al*.^32^. Intracellular Mg^2+^ has been shown to regulate the activity of both NBCe1-B and erythrocyte AE1^18,19^. Titrating Mg^2+^ into ntcNDCBE had no effect on tryptophan fluorescence, nor did 1 mM Mg^2+^ influence the response of ntcNDCBE to Zn^2+^. This result shows that the H167-X-H169 motif does not recognize Mg^2+^, supporting the specificity for Zn^2+^ of this site. However, ITC studies suggest that two high-affinity Mg^2+^ sites are present elsewhere in ntcNDCBE. Despite the relatively low Zn^2+^ affinity compared with free intracellular concentrations, the observation that Zn^2+^ binding is conserved in the ntcNBCs argues that it is not a sporadic event. Recently, numerous studies have focused on elucidating the role of Zn^2+^ not only as a structurally and catalytically important co-factor but also as a signalling molecule^25,30^. Owing to the broad tissue distribution of NBCs in the *SLC4* family, the observed micromolar Zn^2+^-binding affinity could potentially be a general feature in tissues throughout the body^33^. With regard to Zn^2+^ signalling in the brain, it is of particular interest that NDCBE was recently proposed to primarily localize in the pre-synaptic vesicles of glutamatergic neurons and not in the plasma membranes of neurons^6^. Neurotransmitter uptake in presynaptic vesicles by the glutamate transporter VGLUT depends on the electrochemical potential and Cl^−^ concentration^34^. This necessary Cl^-^ uptake has been suggested to be carried out by NDCBE or CLC-3^6^. Furthermore, NDCBE activity depends on a Na^+^ gradient. The Na^+^ gradient is established by NHE6, which has been shown to localize to presynaptic vesicles, where it exchanges vesicular H^+^ for cytoplasmic Na^+^ or K^+^ in an electroneutral transport mode^35^. Zn^2+^ has been shown to accumulate in presynaptic vesicles of glutamatergic neurons. Efflux from the cytoplasm to the vesicle is facilitated by the Zn^2+^ exporter ZnT-3^36^. However, how Zn^2+^ is bound and transported by ZnT-3 is currently unknown, although the reported putative Zn^2+^-binding region (a histidine-rich stretch) has a predicted affinity in the low micromolar range^37^. This affinity is in the same range as the identified Zn^2+^-binding site of ntcNDCBE, which suggests the possibility of a specific role for the observed interaction between Zn^2+^ and the N-terminal cytoplasmic domain of NDCBE in presynaptic vesicles. The impact of Zn^2+^ on NCBE activity suggests that the effect of Zn^2+^ binding is to reduce NBC activity. This interpretation was supported by the finding of an inhibitory effect of 10 µM Zn^2+^ on recovery from acidosis in smooth muscle cells of rat mesenteric small arteries most likely mediated by an inhibitory effect on NBCn1.

In conclusion, Zn^2+^ binding to the regulatory domain of NBC’s suggests an overall regulatory role of Zn^2+^ on membrane transport of acid/base equivalents. In the case of NDCBE, the Zn^2+^ binding may aid in the ion transfer across the presynaptic membrane and thus play a role in neuronal signalling. The inherent pH dependence of this Zn^2+^ site may also reflect the site of pH sensitivity present in the NBCs. As small concentrational variations in intracellular Zn^2+^ would affect the equilibrium between the histidine site and free Zn^2+^ ions, and this would consequentely act as a pH sensor.

The dimer interface presented here only allows two possible sites of interaction with the transmembrane domain, and based on a multiple sequence alignment of the human AEs and NBCs, we have described the conservation of the ntcNDCBE structure. Based on the assumption that the highest conservation is often clustered at the core of the protein fold or at functional sites^21^, we propose the most likely orientation of this domain in relation to the transmembrane domain. We propose that the interface of the N-terminal cytoplasmic domain that interacts with the transmembrane domain is on the opposite side of the dimer compared with that suggested by Gill *et al*. for ntcNBCe1-A^38^. This previous suggestion was based on the localization of three lysine and three leucine residues. Of these six residues from NBCe1-A, only Leu152 and Lys157 (as arginine) are conserved. The N-terminal domain residues responsible for the interaction will most likely not interact with the membrane itself but with residues in the transmembrane domain. In the proposed model of NDCBE (Fig. 6b), the dimer is presented as domain swapped compared with the transmembrane dimer domain. Models of both the swapped and parallel organizations of the N-terminal relative to the transmembrane domain have been proposed previously^39^. The orientation of the two central β-strands and the localization of the first residues of the transmembrane domain of AE1^10^ point towards the domain-swapped hypothesis, though no experimental data are currently available to either confirm or reject this idea. As such, the proposed model is intended merely to guide future experiments on this class of proteins, whose structures are notoriously difficult to obtain.

## Methods

All chemicals were purchased from Sigma-Aldrich unless otherwise stated.

### Ethical approval

All experiments conformed to guidelines from European Convention for the Protection of Vertebrate Animals used for Experimental and other Scientific Purposes and were approved by and conducted with permission from the Animal Experiments Inspectorate of the Danish Ministry of Environment and Food.

### Cloning, expression and purification

Codon-optimized pET-M11 plasmids encoding the N-terminal core (amino acids 93–426) of human NCBE (SLC4A10) isoform B (uniprot ID: **Q6U841-2**) and the complete N-terminal core domain (amino acids 40–425) of human NDCBE (SLC4A8) isoform D (uniprot ID: **Q2Y0W8-4**) were purchased (GenScript). The Quikchange Lightning Site-Directed Mutagenesis kit (Agilent) was used to mutate the ntcNDCBE constructs used in this study, yielding ntcNDCBE (amino acids 40–344), ntcNDCBE_H167A, ntcNDCBE_H169A, ntcNDCBE_H167A, H169A and ntcNDCBE_W80F. The ntcNCBE and ntcNDCBE constructs were N-terminally tagged with a hexa-histidine tag and TEV protease cleavage site, which were present in the vector. *E. coli* BL21 Gold (Agilent) was used as the expression strain for ntcNDCBE and ntcNCBE constructs. The cells were grown by fermentation in terrific broth (TB) medium supplemented with 25 µg/mL kanamycin to an OD_600_ of 2.0 at 37 °C. Protein expression was induced with 0.3 mM IPTG at 18 °C O/N. The cells were harvested by centrifugation at 48,000 × *g*. All subsequent steps were carried out at 4 °C. Cells were lysed in buffer A (100 mM NaCl, 100 mM Tris pH 7.2, 1 mM PMSF, 5 mM β-mercaptoethanol (β-ME) and 1 µg/mL DNase I) using an Emulsiflex high-pressure homogenizer (Avestin). The lysate was cleared by centrifugation at 48,000 × *g* for 20 min and applied to a HisTrap HP column (GE Healthcare) pre-equilibrated in buffer B (100 mM NaCl, 100 mM Tris pH 7.2, 40 mM imidazole and 5 mM β-ME). The protein was eluted with buffer C (buffer B supplemented with 300 mM imidazole) and dialyzed overnight against buffer B in the presence of TEV protease (molar ratio, 50 construct:1 TEV) against buffer B to cleave the his-tag. The cleaved construct was loaded onto an IMAC column pre-equilibrated in buffer B. The flowthrough fractions were concentrated to 10 mg/mL (10-kDa MWCO spin filter, Sartorius) and subjected to SEC on a Superdex S200 column (GE Healthcare) equilibrated with buffer D (100 mM NaCl, 20 mM Tris pH 7.2 and 2 mM DTT). The peak fractions were pooled and concentrated to 10 mg/mL. The constructs were treated with EDTA (molar ratio, 1 construct:5 EDTA) and dialyzed against buffer E (5 mM Tris, pH 7.2, 100 mM NaCl and 0.2 mM TCEP) for use in downstream experiments. Purification was evaluated by SDS-PAGE.

### Crystallization, data collection and structure determination

Purified ntcNDCBE was crystallized via sitting-drop vapour diffusion at 4 °C (40 mg/mL) with 0.1 M formic acid, pH of 4.4, and 9% w/v PEG 8000 and was cryo-protected with 20% ethylene glycol. X-ray diffraction data were collected from a single crystal at the BESSY II 14.1 beamline. The data were processed using XDS^40^ in XIA2^41^. Molecular replacement (MR) was performed with phaser in CCP4^42^. A truncated version of the cdb3 crystallographic model (PDB ID: **1HYN**)^16^ was used as the MR search model. The model was built using Coot^43^ and refined in PHENIX^44^. The final data collection and refinement statistics are presented in Supplementary Table S1.

### Tryptophan fluorescence

Purified, EDTA-treated ntcNDCBE and ntcNCBE were diluted in 20 mM BIS-TRIS and 100 mM NaCl to a final concentration of 2 µM, and intrinsic tryptophan fluorescence was measured at the indicated pH values (6.8–7.2). The titrants used were ZnCl_2_ (Fisher Scientific), MgCl_2_, CaCl_2_ and EDTA. The measurements were performed in triplicate on a Jasco FP8500 spectrofluorometer at 25 °C with a stirring speed of 200 rpm and a 30 sec delay between injection and measurement. The cell path length was 10 mm. The excitation wavelength was 295 nm, and emission spectra were recorded from 310 nm to 500 nm. Data were corrected for the dilution factor. The increase in tryptophan fluorescence (F) yield was calculated at the wavelength corresponding to the maximum intensity of the fully Zn^2+^-saturated curve (336 nm). The increase in tryptophan fluorescence yield due to Zn^2+^ titration was calculated using equation (1):

1 $$Y=\frac{F}{{F}_{bound}}+F-{F}_{free},$$

where F_bound_ is F when the construct is fully saturated, and F_free_ is F without any metal added. The decrease in tryptophan fluorescence yield due to EDTA titration was calculated using equation (1); however, F_free_ is F when all metal was chelated. When applicable, curve fitting of the Zn^2+^ titrations were performed with GraphPad using equation (2):

2 $$-{\rm{Y}}=\frac{Bx{X}^{h}}{{K}_{D}^{h}+{X}^{h}}.$$

Curve fitting of EDTA titrations was performed with GraphPad using the modified (repressive) Hill equation, equation (3):

3 $$Y=\frac{Bx{K}_{D}^{h}}{{K}_{D}^{h}+\,{X}^{h}},$$

where B is the maximum value of F, h is the Hill coefficient, and *K*_D_ is the dissociation constant.

### Isothermal titration calorimetry

Purified, EDTA-treated ntcNDCBE and ntcNCBE were dialyzed against buffer containing 100 mM NaCl, 20 mM BIS-TRIS pH 7.2, and 0.2 mM TCEP. The affinities of the constructs for Zn^2+^ were determined by ITC in a MicroCal ITC_200_ system (GE Healthcare) at 25 °C. The sample cell was filled with 200 µL of degassed protein solution at 50 µM. The titration solution of 0.9–1.5 mM metal in dialysis buffer was titrated in 2 µL injections with a 90-sec delay between injections. Background subtraction was performed using the appropriate buffer. Data integration and curve fitting were performed with the Origin ITC analysis package. ITC experiments were repeated at least twice for each construct, with similar results in both the number of sites and the affinity range.

### Alignment and conservation mapping

Representative isoforms of each human NBC and AE of the SLC4 family were aligned using MUSCLE^45^. Based on sequence conservation, ntcNDCBE was divided into VR1 (1–39); CR1 (40–128); VR2 (129–236); and CR2 (237–344). The resulting alignment was mapped onto ntcNDCBE and the transmembrane domain of AE1 (PDB ID: 4YZF, ref.^10^) using the online server ProtSkin^46^. The residue conservation score was based on sequence identity.

Uniprot identifiers: NDCBE – **Q2Y0W8-4**; NBCn1 – **Q9Y6M7-1**; NCBE – **Q6U841-1**; NBCe1 – **Q9Y6R1-1**; NBCe2 – **Q9BY07-1**; AE1 (**1HYN**/**4KY9**) – **P02730-1**; AE2 – **P0492-1**; AE3 – **P48751-1**.

### Intracellular pH measurements in *slc4a10*-transfected cells

*Slc4a10*-transfected NIH-3T3 fibroblasts were previously described^47^. The cells were stably transfected with a plasmid containing the full length mouse *slc4a10* construct (corresponding to rb1Ncbe, AB033759). Cells were cultured in Dulbecco’s modified Eagle’s medium glutaMAX^TM^ supplemented with 10% donor bovine serum. For pH_i_ measurements cells were grown on glass coverslips.

Prior to measurements the cells were loaded with 2 µM BCECF-AM (ThermoFisher scientific) in a HEPES-buffered salt solution for 10 minutes at 37 °C and mounted in a closed perfusion chamber (Warner Instruments). The chamber was perfused with a linear flow rate of ~0.8 mm/s at 37 °C and placed on an inverted microscope stage in a 37 °C dark box. The fluorophore was excited with alternating 495-nm and 440-nm light from a monochromator (Till photonics). The light emission at 510–535 nm was recorded by a 12-bit cooled monochrome CCD camera (QImaging, Retiga EXi) and data was collected from regions of interest of a minimum of 3 cells per coverslips after background correction. Cells were allowed to equilibrate to a baseline level in HEPES buffered salt solution (HBS) followed by acidification by a 20 mM NH_4_Cl prepulse for 5 minutes followed by a Na^+^ free bicarbonate buffered salt solution (Na^+^ free bicarbonate buffered salt (BBS)). The Na^+^ dependent HCO_3_^−^ transport was determined as the dpH_i_/dt after switching to a bicarbonate buffered Na^+^ containing solution (BBS) containing 10 µM EIPA to inhibit Na^+^/H^+^ exchange activity. Each measurement ended in a one-point calibration at pH 7.0 in a high K^+^-solution containing 10 µM nigericin. In separate experiments, the fluorescence ratio was calibrated to pH by clamping pH_i_ stepwise from 6–7.5 in a high K^+^ HBS with 10 µM nigericin^48^.

The solutions contained (in mM): **HBS**: 145 Na^+^, 3.6 K^+^, 1.8 Ca^2+^, 0.8 Mg^2+^, 139 Cl^−^, 0.8 SO_4_^2−^, 5.5 glucose, 10 HEPES, 2 HPO_4_^2−^; **20 mM NH**_**4**_ **Cl solution**: 20 NH_4_Cl, 125 Na^+^, 3.6 K^+^, 1.8 Ca^2+^, 0.8 Mg^2+^, 139 Cl^−^, 0.8 SO_4_^2−^, 5.5 glucose, 10 HEPES, 2 PO_4_^2−^; **Na** ^**+**^ **free BBS**: 24 HCO_3_^−^, 24 choline, 121 N-Methyl D glucamine, 3.6 K^+^, 1.8 Ca^2+^, 0.8 Mg^2+^, 115 Cl^−^, 0.8 SO_4_^2−^, 5.5 glucose, 10 HEPES, 2 HPO_4_^2−^; **BBS**: 24 HCO_3_^−^ 145 Na^+^, 3.6 K^+^, 1.8 Ca^2+^, 0.8 Mg^2+^, 115 Cl^−^, 0.8 SO_4_^2−^, 5.5 glucose, 10 HEPES, 2 PO_4_^2−^. All solutions were adjusted to pH 7.4. Bicarbonate buffers were bubbled with 5% CO_2_. **High K** ^**+**^ **HBS**: 10 Na^+^, 139 K^+^, 1.8 Ca^2+^, 0.8 Mg^2+^, 139 Cl^−^, 0.8 SO_4_^2−^, 5.5 glucose, 10 HEPES, 2 HPO_4_^2−^.

### Intracellular pH measurements in rat mesenteric small arteries

pH_i_ of rat mesenteric small arteries was measured as described previously^49,50^. Eleven week old rats were killed by CO_2_ inhalation where after a segment of a rat mesenteric small artery (internal diameter about 200 µm) was dissected free and mounted in a myograph for isometric force recording. The arteries were kept in a solution (PSS) containing (in mM): Na^+^ 143, 4 K^+^, HCO_3_ 25, 1.6 Ca^2+^, 1.0 Mg^2+^, Cl 130, 2 HPO_4_^2−^ 2.0, 10.0 HEPES, glucose 5.0). The solution was gassed with 5% CO_2_ in air and the temperature kept at 37 °C. VSMC pH_i_ was measured using wide-field microcopy of arteries loaded with 5 µM BCECF-AM (Invitrogen) for 30 min. The preparation was placed on the stage of a microscope and the fluorophore excited with alternating 435 nm and 488 nm light. The emission at 510–535 nm at each excitation wavelength was recorded by a photometer and the ratio of the emissions corresponding to excitation at 488 and 435 nm determined. Intracellular acidification was induced by adding 15 mM NH_4_Cl for 15 min followed by washout into a Na^+^ free PSS (NaCl was substituted with equimolar amounts of N-methyl-D-glucamine titrated with HCl while NaHCO_3_ was substituted with equimolar amounts of choline bicarbonate). After 5 min Na^2+^ was washed in (the Na^+^ free PSS substituted with PSS) and the rate of increase of the emission ratio was taken as a measure of pH recovery. This value was determined under control conditions, in the presence of 10 µM Zn^2+^ and again after washout of Zn^2+^. The pH recovery in the presence of Zn^2+^ was compared to the mean pH recovery in the control condition and after washout of Zn^2+^.

## Electronic supplementary material

Supplementary data
