# Supplementary material for: The crystal structure of the regulatory domain of the human sodium-driven chloride/bicarbonate exchanger
Source: Sci Rep. 2017 Sep 21;7:12131. doi: 10.1038/s41598-017-12409-0 (PMC5608694; doi:10.1038/s41598-017-12409-0)
Supplement: Supplementary file 1 — Supplementary data [file 41598_2017_12409_MOESM1_ESM.pdf]

Title: The crystal structure of the regulatory domain of the human sodium-driven chloride/bicarbonate exchanger

Running title:

Crystal structure of NDCBE

Authors:

Carolina M. Alvadia[1†], Theis Sommer[1†], Kaare Bjerregaard-Andersen[1†], Helle Hasager Damkier[3], Michele Montrasio[1], Christian Aalkjaer[3], J. Preben Morth[1,2*]

1. Norwegian Centre for Molecular Medicine, Nordic EMBL Partnership University of Oslo, Gaustadalléen 21, 0349 Oslo, Norway.

2. Institute for Experimental Medical Research, Oslo University Hospital, N-0424 Oslo, Norway.

3. Department of Biomedicine, Aarhus University, 8000 Aarhus, Denmark.

[†] Shared first author.

*Corresponding author. E-mail: j.p.morth@ncmm.uio.no

Supplemental information– Figures

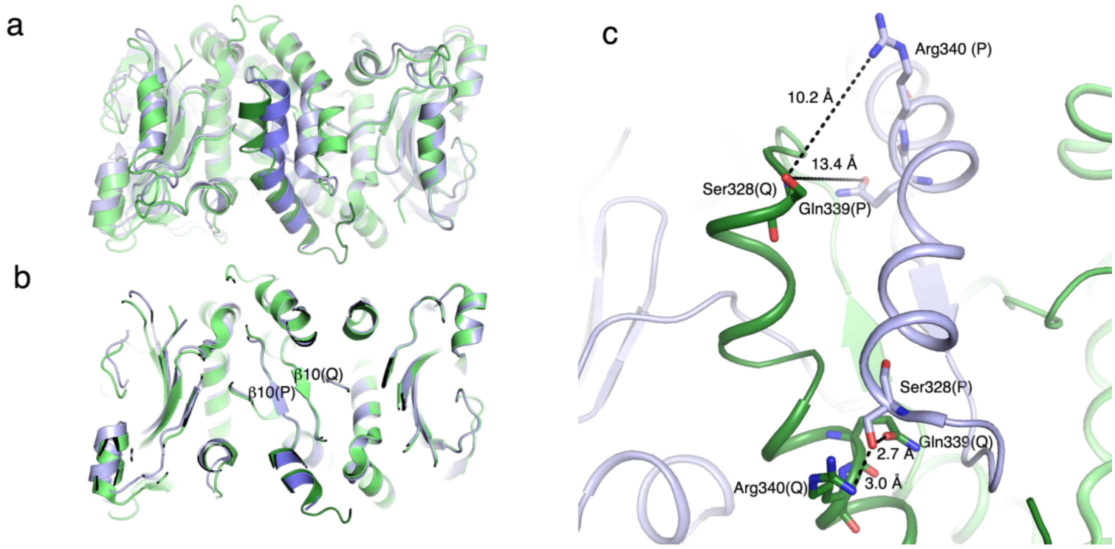

Supplementary Figure S1 **Asymmetry observed between the dimerization helix in cdb3**

(a) The chains Q(Q) and P(P) in the crystal structure of cdb3 (PDB ID 1HYN), form a dimer. Superimposition of chain P (light blue) with chain Q (light green) show that the strongest symmetric deviation is found at the dimer interface between the dimerization helix spanning residues 327 to 356 in both chains. The dimer helix is highlighted by dark green for chain Q and dark blue for chain P. **(b)** The dimer interface that include the domain swapped beta chain for both chain P and Q and labelled β10 is structurally conserved. **(c)** A zoomed view of the dimer helix interface. As a consequence of symmetrical deviation, the hydrogen bonding network or hydrophobic interactions that stabilize the dimer interface is not present. This is exemplified by, the bifurcated hydrogen bond that exist between the hydroxyl group of Ser328 in chain P to the sidechains of Gln339 and Arg340 of chain Q. This hydrogen bond network cannot form in the equivalent Ser328 in chain Q to Arg340 and Gln339 in chain P. Maintaining a symmetrical geometrical arrangement is known to be under strong selection pressure [1] and indeed a common parameter used for defining dimer interfaces, is that a dimer interface maintains local geometry[2].

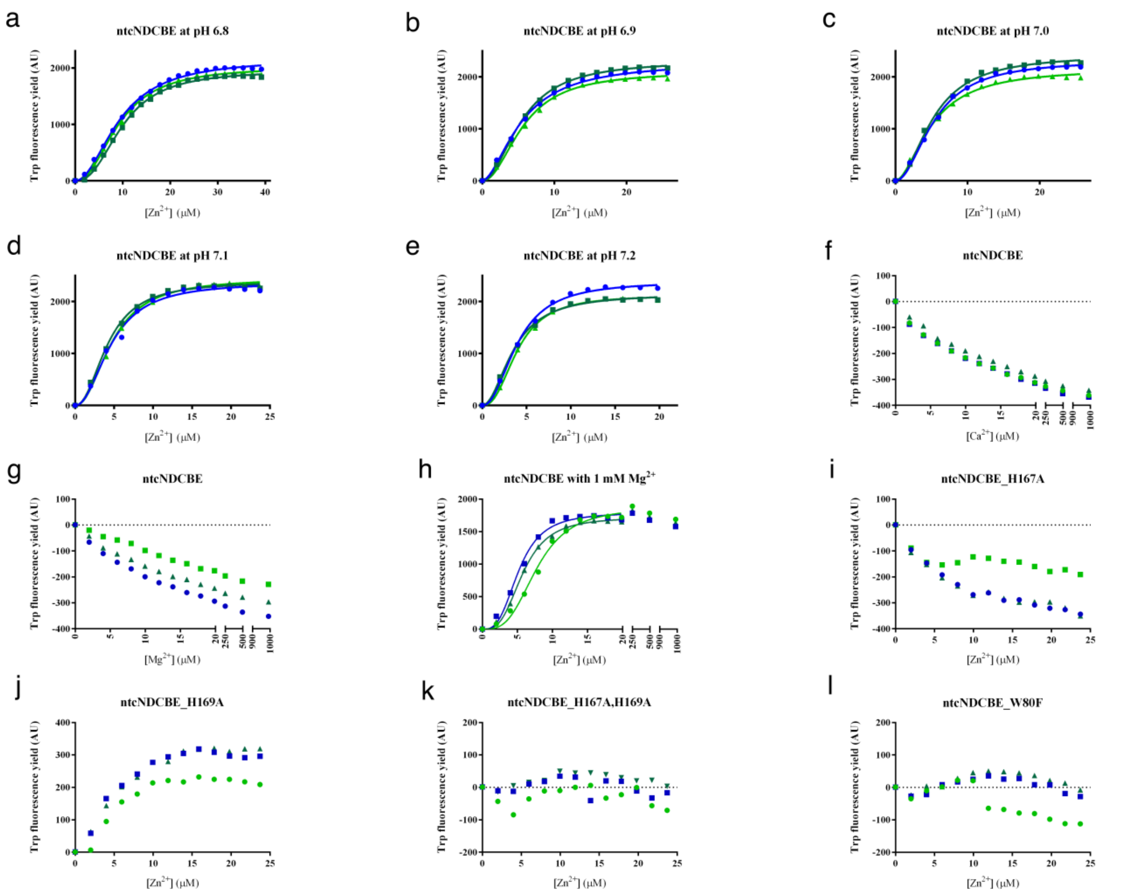

Supplementary Figure S2 **Effect of metal titration on tryptophan fluorescence yield of ntcNDCBE.** Raw data of the three measurements of tryptophan fluorescence yield of ntcNDCBE **(a)** at pH 6.8. **(b)** at pH 6.9. **(c)** at pH 7.0. **(d)** at pH 7.1. **(e)** at pH 7.2. **(f)** Raw data of the three measurements of tryptophan fluorescence yield when titrating Ca^{2+} into ntcNDCBE at pH 7.2. **(g)** Raw data of the three measurements of tryptophan fluorescence yield when titrating Mg^{2+} into ntcNDCBE at pH 7.2. **(h)** Raw data of the three measurements of tryptophan fluorescence yield when titrating Zn^{2+} into ntcNDCBE at pH 7.2 in the presence of 1 mM Mg^{2+}. **(i)** Raw data of the three measurements of tryptophan fluorescence yield at pH 7.2 when titrating Zn^{2+} into (I) ntcNDCBE_H167A. **(j)** ntcNDCBE_H169A. **(k)** ntcNDCBE_H167A, H169A. **(l)** ntcNDCBE_W80F. The fits (lines) were calculated with equation (2).

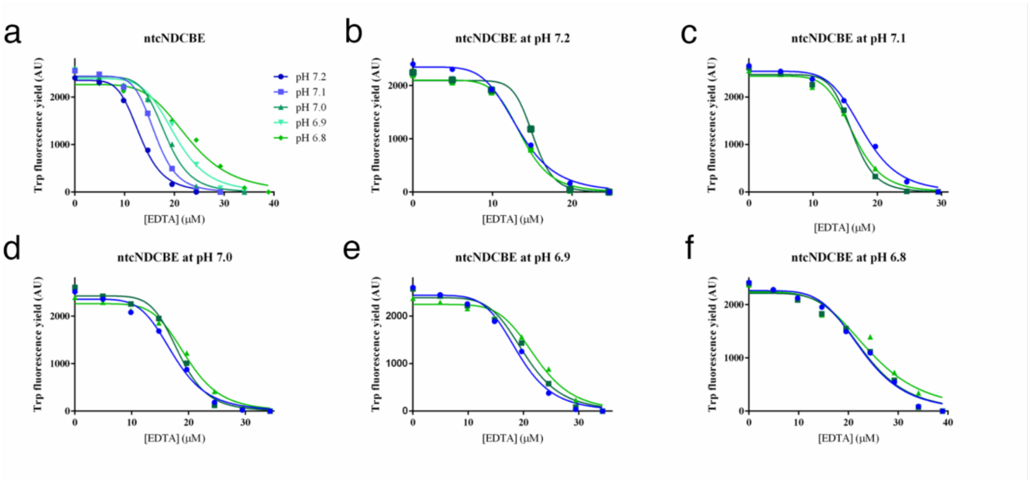

Supplementary Figure S3 **Effect of EDTA on tryptophan fluorescence yield of fully Zn^{2+}-saturated ntcNDCBE. (a)** Raw data of single measurements of tryptophan fluorescence yield of ntcNDCBE at different pH values. **(b)** at pH 7.2. **(c)** at pH 7.1. **(d)** at pH 7.0. **(e)** at pH 6.9. **(f)** at pH 6.8. The fits (lines) were calculated with equation (3).

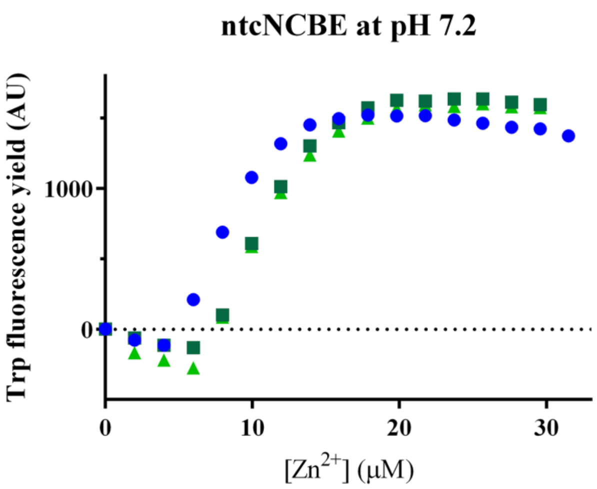

Supplementary Figure S4 **Effect of Zn^{2+} titration on tryptophan fluorescence yield of NBCs.** Raw data of the three measurements of tryptophan fluorescence yield at pH 7.2 of ntcNCBE.

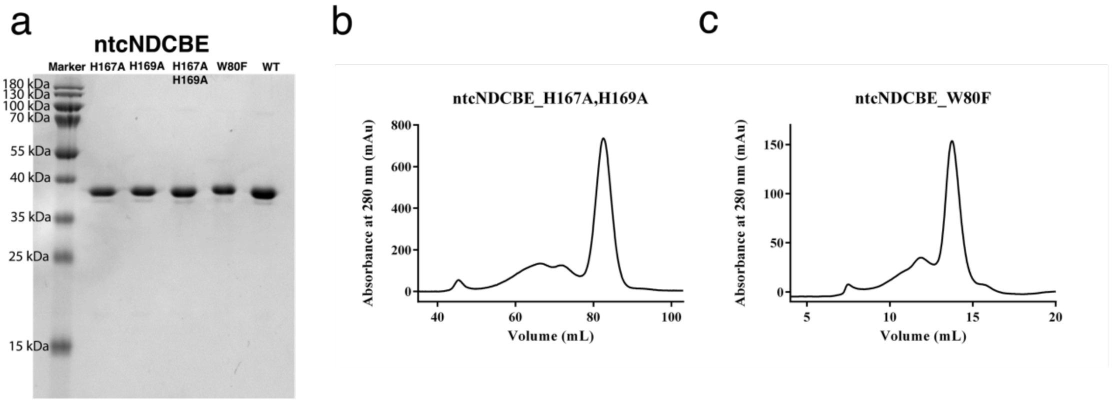

Supplementary Figure S5 **Purity of ntcNDCBE constructs. (a)** SDS-PAGE of the different ntcNDCBE constructs. All the construct run equally. Marker: PageRuler Prestained Protein Ladder (Thermofisher). The gel was coomassie stained. **(b)** SEC run of the double histidine mutant (ntcNDCBE_H167A, H169A). The construct runs as a monodisperse peak with an elution volume equivalent to the wild-type ntcNDCBE. SEC column: Superdex 16/600. **(c)** SEC run of the ntcNDCBE_W80F mutant. The construct runs as a monodisperse peak with an elution volume equivalent to the wild-type ntcNDCBE. SEC column: Superdex 10/300.

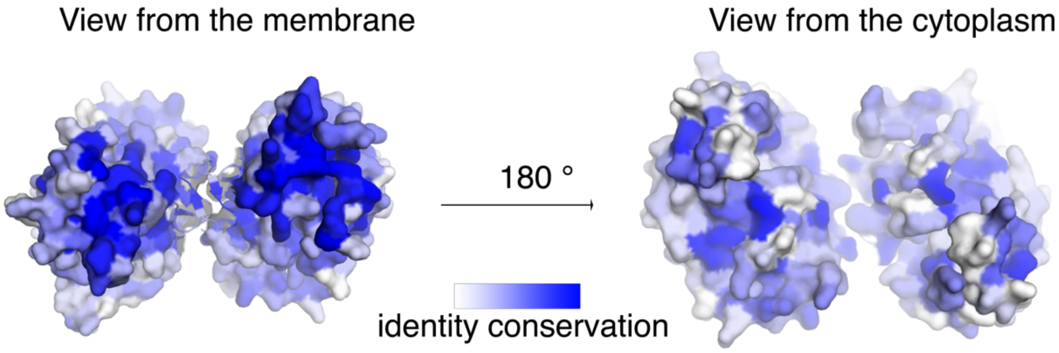

Supplementary Figure S6 **Conservation of AEs and NBCs displayed in the structure of ntcNDCBE.** The dimer forms a two-fold rotational axis perpendicular to the β10/β10B sheet, leaving only two surfaces with identical conserved residues facing the same direction. To identify the most conserved, surface the model was rotated by 180 ° around the x-axis. The conservation level is coloured from white (lowest) to bright blue (highest).

Supplementary information – Tables

	ntcNDCBE (5JHO)
**Data collection**	
Beamline	14.1 BESSY II
Wavelength (Å)	0.918409
Space group	$P4_322$
Cell dimensions	
a, *b*, *c* (Å)	98.04, 98.04, 212.9
α, β, γ (°)	90, 90, 90
Resolution (Å)	69 - 2.8 (2.87 - 2.8)[a]
R_{merge} (%)	5.3 (>100)
$CC_{1/2}$	1 (0.8)
$I / \sigma I$	30.5 (2.5)
Completeness (%)	99.9 (99.5)
Redundancy	11 (11)
No. reflections	
Total	20624
Unique	1898
**Refinement**	
Resolution (Å)	25 - 2.8
R_{work} / R_{free}	0.200 / 0.238
No. residues	473
No. water molecules	5
Average *B*-factor	99
R.m.s. deviations	
Bond lengths (Å)	0.010
Bond angles (°)	1.4
Ramachandran favoured (%)	96.1
Ramachandran outliers (%)	0
Ramachandran allowed (%)	3.9

[a]Diffraction data was obtained from one crystal.

Supplementary Table S2 **Hill binding parameters of tryptophan fluorescence experiments with ntcNDCBE**

**Experiment**	**Hill coefficient**	K_D **(μM)**	R^2
ntcNDCBE with Zn^{2+} at pH 7.2	2.0 – 2.5	3.6 – 4.0	> 0.99
ntcNDCBE with Zn^{2+} at pH 7.1	2.1 – 2.2	4.2 – 4.8	> 0.99
ntcNDCBE with Zn^{2+} at pH 7.0	1.8 – 2.0	5.1 – 5.4	> 0.99
ntcNDCBE with Zn^{2+} at pH 6.9	1.7 – 2.1	5.5 – 5.7	> 0.99
ntcNDCBE with Zn^{2+} at pH 6.8	2.0 – 2.4	9.3 – 10.0	> 0.99
ntcNDCBE with EDTA at pH 7.2	5.6 – 12.1	13.3 – 15.1	≥ 0.99
ntcNDCBE with EDTA at pH 7.1	6.4 – 9.2	16.1 – 17.8	> 0.99
ntcNDCBE with EDTA at pH 7.0	5.7 – 7.3	17.4 – 19.7	≥ 0.99
ntcNDCBE with EDTA at pH 6.9	6.0 – 6.5	19.0 – 22.3	≥ 0.98
ntcNDCBE with EDTA at pH 6.8	4.4 – 5.1	22.8 – 24.3	≥ 0.96

References

1. Marianayagam, N. J., Sunde, M. & Matthews, J. M. The power of two: protein dimerization in biology. *Trends Biochem. Sci.* **29**, 618-625 (2004).

2. Perica, T., Chothia, C. & Teichmann, S. A. Evolution of oligomeric state through geometric coupling of protein interfaces. *Proc. Natl. Acad. Sci. U. S. A.* **109**, 8127-8132 (2012).
